# An Over-the-Counter Hearing Aid Clinical Trial in Rural Alabama: Project Design and Potential Implications for Pharmacy and Audiology Interprofessional Collaborations

**DOI:** 10.3390/pharmacy12030076

**Published:** 2024-05-11

**Authors:** Marcia J. Hay-McCutcheon, Abigail F. Hubbard, Emma B. Brothers, Rebecca S. Allen, Xin Yang

**Affiliations:** 1 Department of Communicative Disorders, The University of Alabama, Tuscaloosa, AL 35487, USA; aflesley@ua.edu (A.F.H.); emma.brothers@ua.edu (E.B.B.); xyang15@ua.edu (X.Y.); 2 Alabama Life Research Institute, The University of Alabama, Tuscaloosa, AL 35487, USA; rsallen@ua.edu; 3 Alabama Research Institute on Aging, The University of Alabama, Tuscaloosa, AL 35487, USA; 4 Department of Psychology, The University of Alabama, Tuscaloosa, AL 35487, USA

**Keywords:** hearing aids, rural health, community health, healthcare quality, aging

## Abstract

Over-the-counter hearing aids (OTC HAs) have the potential to help adults with perceived mild-to-moderate hearing loss across the US, especially in rural communities, where access to hearing healthcare is extremely limited or non-existent. The purpose of this study was to describe an OTC HA clinical trial being conducted in five rural counties of Alabama and to provide preliminary anecdotal data related to the use and care of these hearing aids by the participants. In brief, for this clinical trial, adults with hearing loss were randomly placed in one of three groups where they received varying levels of support for setting, using, and maintaining their OTC HAs. Listening tests and surveys were administered to assess the extent to which they benefitted from the hearing aids as related to word understanding, communication with others in natural settings, and hearing aid use and care. Currently, anecdotal findings suggested that, although some participants required very little support to successfully use their hearing aids, others had difficulty setting and caring for their devices and could have benefitted from individualized guidance. Future quantitative studies will assess the extent of support needed for successful hearing aid benefit and use. Potentially, collaborations among pharmacy and audiology professionals could lead to increased access to hearing healthcare by supporting the use and purchase of OTC HAs in rural pharmacy settings.

## 1. Introduction

The 2022 FDA Final Rule for Over-the-Counter Hearing Aids (OTC HAs) has the potential to revolutionize access to hearing aids by allowing adults with perceived mild-to-moderate hearing loss to purchase these devices over the counter, without medical clearance or care from an audiologist [1,2]. Estimates suggest that approximately 25 million adults over the age of 18 years suffer from mild-to-moderate hearing loss in the United States (US) [3] and these individuals now have the option to address their own hearing loss. The FDA rule has allowed people to purchase these devices at supermarkets, pharmacies, consumer electronic stores, or online, thereby potentially increasing access to hearing healthcare. The use of hearing aids in older populations has been shown to enhance social contact, eliminate loneliness, and have a positive impact on cognition [4,5,6,7]. However, current evidence suggests that, since their release in October 2022, only about 2% of adults with perceived mild to moderate hearing loss have purchased OTC HAs [8]. Understanding how to improve the uptake of OTC HAs could help mitigate the negative effects of hearing loss, especially in rural communities where there are few, if any, hearing healthcare resources [9]. Considering the availability of local community pharmacies in rural US settings, where approximately 60% of adults in rural communities have access to a chain pharmacy and 16% have access to an independent pharmacy [10], the potential to develop interprofessional collaborations between pharmacy and audiology professionals could lead to improved hearing healthcare for adults with hearing loss in these settings. 

In fact, Berenbrok, Mormer, and colleagues have reported on collaborations in their recent work [11,12,13,14,15]. These researchers, representing pharmacy and audiology professions, have developed 26 competency statements for pharmacists when providing hearing healthcare in pharmacies [14], developed online training sessions designed to help pharmacists become familiar with supporting adults with hearing loss [13], and reported findings from a patient who received support from a pharmacist in the provision of OTC HAs [15]. Evidence has also indicated that pharmacists are very interested in working with audiologists to support adults with hearing loss [11,12,16]. Brothers and colleagues used focus group discussions and individual interviews to reveal that pharmacists were interested in collaborating with audiologists and other hearing healthcare professionals to increase access to hearing healthcare. However, pharmacists have expressed concerns that, currently, they lack the knowledge to provide support and that, with limited time and staff available, an additional service may not be feasible in certain pharmacies [11,12,16]. 

Prior evidence has suggested that adults can significantly benefit from hearing aids provided through a consumer-driven model where they select and fit their own devices [17,18,19]. Specifically, word understanding, social interactions, and emotional well-being significantly increased after these consumer driven devices were provided. Outcomes also demonstrated that when adults selected their hearing aids using a consumer-driven model they reported less satisfaction with their hearing aid compared to adults who received audiological support when selecting and fitting an aid [17,18]. Overall, these results provide evidence that a consumer driven model of hearing aid fitting is effective, and that initial audiological care helps to orient the consumer and promote successful use of the device.

It is important to note that these prior studies were conducted in urban higher educational settings [17,19]. No research is currently available outlining the success of OTC HA use in rural, underserved communities where there is little or no support for these devices. One of the goals of the National Academy of Sciences, Engineering, and Medicine (NASEM) 2016 report, Hearing Health Care for Adults: Priorities for Improving Access and Affordability [20], is to improve hearing healthcare access for underserved and vulnerable populations. Consequently, to address this goal, further study will be required in underserved rural regions of the country.

Specifically, it will be necessary to examine how residents in rural communities deal with aspects of OTC HA purchase and use [9,21]. These consumers must self-identify their mild-to-moderate hearing loss and ensure that they have no other ear disorders that are contraindicative for hearing aid use. Additionally, users will need to learn how to set their devices, how to care for and manage their hearing aids, and understand how to troubleshoot them when they are not working. These issues could be barriers to optimal benefit from OTC HAs [21]. Potentially, as suggested by Berenbrok and others, health care professionals, including pharmacists and pharmacy technicians, could be trained to provide basic hearing healthcare in these underserved communities to help mitigate the effects of hearing loss and improve access to hearing healthcare [11,14,15,21]. 

Considering the availability of OTC HAs through big box stores, pharmacies, and online sources, and their potential to increase accessibility for those with hearing loss across the US, it will be important to assess barriers to use. The purpose of this study was to provide an outline of a clinical trial study (ID: NCT04671381) taking place in rural communities of Alabama where residents were randomly placed in one of three groups and provided with varying levels of support after receiving bilateral OTC HAs. Additionally, preliminary anecdotal information related to initial use and maintenance of the OTC HAs for 51 participants placed in these groups was reported. 

## 2. Materials and Methods

### 2.1. Project Design

An outline of the procedures associated with the clinical trial is provided in Table 1. This trial took place over a 14-week period and contained six different appointments associated with hearing evaluations, setting OTC HAs, and evaluating their use. Additionally, each participant attended four weeks of one-hour sessions of information related to hearing loss (i.e., aural rehabilitation) and four weeks of one-hour general health sessions (i.e., nutrition, exercise, cardiovascular health, and emotional well-being). Further details of the clinical trial are provided below.

### 2.2. Over-the-Counter Hearing Aids

Over-the-counter HAs (HD75R) from Sound World Solutions (Park Ridge, IL) were used for this study. These FDA-registered, open-fit hearing aids have the receiver in the ear canal (RIC). There are two buttons on these behind-the-ear devices that control the power, volume, gain presets, and environmental listening modes. These aids are equipped with three gain presets (i.e., 1, 2, and 3) for varying degrees of hearing loss and three listening modes (everyday, restaurant, and entertainment). The hearing aids arrived from the manufacturer on preset 2 and the listening environment was set to “everyday.” The hearing aids were provided to study participants free of charge.

### 2.3. Hearing Evaluation and Survey Completion (Appointment 1)

Appointment 1 took place during the first week of the trial and consisted of a hearing evaluation where behavioral thresholds for tones and speech were obtained and participants completed two listening tests, one in a quiet environment and a second in a noisy environment. Also, participants completed surveys on an iPad using the Research Electronic Data Capture (REDCap) software program (14.0.26) hosted by the University of Alabama [22,23]. Two audiologists (A1 and A2) conducted the hearing evaluations and a different research assistant helped with survey completion, if needed, by reading the questions to the participants or selecting their answer on the iPad for them. Informed consent was obtained prior to the hearing evaluation and survey completion. A demographic form also was completed before the hearing testing. After this appointment, participants were randomly placed in one of three groups, Auditory Best Practices with Aural Rehabilitation (ABP+AR), ABP, and Over-the-Counter hearing aids only (OTC-Only). More specific information about these three groups and the tasks completed by each group are provided below.

The primary measures, or surveys, for the clinical trial included the Hearing Handicap Inventory for Elderly (HHIE) [24] and the International Outcome Inventory for Hearing Aids (IOI-HA) [25]. Twenty-five questions of the HHIE measure emotional and social/situational consequences of hearing loss, including feelings of embarrassment, social isolation, and frustration. Participants answer each question using the option of “Yes” (4 points), “Sometimes” (2 points), and “No” (0 points). Higher scores are indicative of a greater hearing handicap. The IOI-HA is a seven-item inventory that assesses daily HA use, benefit, activity limitation, satisfaction, participation limitation, impact on others, and quality of life. Responses to each question are provided using a 5-point rating scale tailored to each question. The maximum score is 35 and higher scores suggest better outcomes with hearing aids. 

Two listening tests were administered by an audiologist to assess word understanding in quiet and in noise. Both tests were presented in a sound-treated booth. The Northwestern University test (NU-6) [26] is an open-set test where words are presented in a sound treated booth and listeners repeat what they hear. A percentage correct word understanding is obtained for each participant. The second listening test, the QuickSIN, is a sentence understanding test where participants repeat sentences in the presence of background noise presented from a speaker [27]. A signal-to-noise ratio (SNR) is obtained, which is the difference in the intensity levels between the signal and the noise. Two different sentence lists were administered. Both listening tests were secondary measures for the clinical trial.

Other secondary measures included the Client Oriented Scale of Improvement (COSI) [28], the Abbreviated Profile of Hearing Aid Benefit (APHAB) [29], and the World Health Organization Quality of Life–Age (WHOQOL-AGE) survey [30]. The COSI is a 16-item, clinician-administered survey that addresses how individuals’ five most important listening difficulties have been alleviated with OTC HA use, using a 5-level rating scale. The APHAB is a 24-item survey that computes OTC HA benefit by calculating the difference between aided and unaided conditions and uses a 7-point rating scale. Finally, the WHOQOL-AGE is a 13-item quality-of-life measure for older adults using a rating scale of 5-levels. Generally, the questions assess physical health, personal relationships, finances, living space, and activities of daily living. 

Two control measures, the Patient Health Questionnaire–9 (PHQ-9) [31] and the Charlson Comorbidity Index [32] were completed. Outcomes from these measures provided information related to states of physical and emotional well-being that could have influenced the outcomes of the primary and secondary measures. The PHQ-9 is a nine-question screening tool used to assess signs of depression and was administered to evaluate potential emotional conditions that could impact the outcomes. The rating scale ranges from 0 (“not at all”) to 3 (“nearly every day”) for each question and a score of 10 or above has been used to identify possible depression [33]. The Charlson Comorbidity Index was used to assess the presence of other physical conditions such as heart disease, cancer, liver, kidney disorders, ulcers, and diabetes, to name a few.

### 2.4. Hearing Aid Setting (Appointment 2)

During Appointment 2, the ABP+AR and the ABP groups had their OTC HAs provided by an audiologist (A1), where they were set to best match the participants’ hearing loss. Both groups also received an OTC HA orientation where they were given information about each feature of the hearing aid. Participants in the ABP group received the same services from the audiologist as those in the ABP+AR group, but they did not receive four weeks of AR. Finally, participants in the OTC-Only group did not have their OTC HAs set by the audiologist. Rather, the audiologist gave them two OTC HAs in a box along with a small-print, 26-page manufacturer User Guide, outlining the directions for setting them. These participants also had the option to view the User Guide in a large-print format on a computer screen. The audiologist left the participant to set the aids on their own and checked on them after 15 to 20 min. If the participant had no difficulty setting the device, they were left on their own to complete the task. If the participant was struggling to understand how to set the devices, the audiologist guided them to the instructions in the User Guide where they could find more information. If they still required assistance, the audiologist demonstrated the feature but did not set the devices. These participants did not receive an OTC HA orientation. This group also attended general health sessions, provided to them separately from those participants in the ABP group. 

The degree of assistance from the audiologist provided to participants in the OTC-Only group was classified as “no guidance,” “minimal guidance,” “moderate guidance,” and “full guidance.” For those needing no guidance, the participant set the device independently without help from the audiologist. For minimal guidance, one or two hearing aid features were demonstrated by the audiologist; for moderate guidance, more than two hearing aid features were demonstrated. Full guidance was given when the participant needed demonstrations for setting all components of the hearing aid. The levels of guidance were determined through discussion between the audiologist providing the OTC HAs to these participants and the study principal investigator.

### 2.5. Aural Rehabilitation (AR) and General Health Sessions

Following Appointment 2, participants either attended four weeks of AR or four weeks of general health sessions. Participants in the ABP+AR group attended the AR sessions conducted by trained Community Health Workers (CHWs), where they received more information about their hearing loss, their hearing aids, and how to address communication breakdowns. Participants in the ABP and OTC-Only groups attended four one-hour general health sessions that were presented by fellow community residents who had medical or educational backgrounds. The topics for these sessions included nutrition, exercise, cardiovascular health, and emotional well-being. Separate presentations of the general health sessions were held for the ABP and OTC-Only groups. Because the OTC-Only group had the option to receive hearing aid support during Appointment 5 (see below), equivalent to what the ABP group received during Appointment 2, both the ABP and the OTC-Only group participants attended the AR sessions together during the second group of educational sessions in weeks 10 to 13 of the clinical trial (see Table 1).

### 2.6. Listening Test and Survey Completion (Appointments 3, 4, and 6)

The listening tests described above were completed one week following receipt of the hearing aids and during Appointments 4 and 6, which occurred after the AR or general health sessions. Audiologist 2 (A2) administered the tasks for Appointments 3 and 4 and Audiologist 3 (A3) administered the tasks for Appointment 6. The use of three audiologists, in addition to the blinded research assistant, prevented bias from being introduced into the outcomes from the listening tests and survey measures. Also, at each of these appointments, the audiologists recorded any unsolicited comments that participants provided and noted the placement of the hearing aid and if it was charged and in working condition. 

### 2.7. Hearing Aid Review (Appointment 5)

This appointment was optional for participants. If they wanted further guidance related to their hearing aid settings or the care and use of the hearing aids, this appointment was available to them. It was originally thought that participants in the OTC-Only group would benefit from more specific information about the OTC HA functions prior to their AR sessions, which was the impetus for offering this appointment. However, it was found that participants in the other two groups also were interested in attending these appointments. During Appointment 5, the hearing aid components and features were reviewed with the participants by A1 or A2. Both A1 and A2 did not administer tests after this appointment.

### 2.8. Qualitative Study

To present preliminary findings related to the use and care of the OTC HAs, anecdotal notes obtained from participants who completed Appointments 2, 3, and 4 were assembled. Unsolicited comments were recorded along with information related to how the hearing aids were being used and maintained. The audiologists did not ask the participants specific questions related to hearing aid use, rather they recorded comments provided freely by the participants and noted if the hearing aids were charged and how they were placed in the ear. If questions were asked, the audiologists referred the participants to the User Guide provided to them during Appointment 2. Participants also were told not to mention details of service they received during Appointment 2.

Participants were recruited through flyers placed in community settings in five rural counties of West Central and South Alabama, community social media notices, and notices in local newspapers. Written informed consent was obtained prior to participation in the study according to IRB Approval (#EX-19-CM-089). Quantitative data from the listening tests and surveys, obtained from the three study groups, will be reported at a future date. These data will help determine the extent of support needed for successful OTC HA use. 

## 3. Results

### 3.1. Participants

Data from a total of 51 participants who were randomly placed in the ABP+AR, ABP, and OTC-Only groups were included in the study. For forty of these participants, notes were obtained for all appointments; for eleven participants, notes were obtained either for Appointment 3 (seven participants) or for Appointment 4 (four participants). They ranged in age from 51 to 91 years of age with a mean of 75 years old and a standard deviation of 8.3 years. Other demographic data are provided in Table 2. Note that perceived income inadequacy, rather than household income, was used as a variable because research has demonstrated that the perception of income is a better indicator of financial strain than household income [34]. Perceived income inadequacy as a measure of financial strain was determined using the question, “How difficult is it for you to pay for the very basics, like food, housing, medical care, and heating?” The response options were, “not difficult at all,” “not very difficult,” “somewhat difficult,” and “very difficult.” 

### 3.2. Anecdotal Comment Organization

Table 3 provides a summary of the notes taken during each OTC HA setting session for participants placed in the OTC-Only group. For these participants, the audiologist did not set the hearing aids. Five of the fifteen participants were able to set their devices without any guidance from the audiologist, four needed minimal guidance where the instructions in the User Guide for one or two hearing aid features were pointed to them, four needed a moderate amount of guidance where more than two features needed to be demonstrated, and four required full guidance where the hearing aid could not be set without the majority of the hearing aid features being demonstrated. Four participants had their sons or daughters accompany them to the appointment. These individuals either read the instructions to their parent, or they attempted to assist with the hearing aid setting. Five participants needed to view the large print version of the User Guide. Other anecdotal notes are provided in Table 3.

Table 4 provides anecdotal comments from participants who attended Appointment 3 where they completed two listening tests, the outcomes of which will be reported at a future date. The audiologist recorded notes related to the placement of the hearing aids and if they were charged when the participants arrived for their appointments. These comments, recorded one week after receiving the OTC HAs, suggested that some participants needed assistance with hearing aid use. Specifically, participants in all groups had questions related to hearing aid use and care (e.g., daily hours of use and proper cleaning), some had difficulty properly charging their hearing aids, some had difficulty placing the hearing aids in their ears, some had technical difficulties with their hearing aids (e.g., hearing aid malfunction or static), and some were not using their hearing aids regularly, if at all. If the hearing aid/s malfunctioned, they were replaced by the audiologist. Finally, several participants across groups, either intentionally or unintentionally, had changed their initial OTC HA presets from their Appointment 2 preset setting.

Table 5 provides a summary of notes recorded from Appointment 4 where participants completed listening tests and surveys. As for Appointment 3, the comments recorded during Appointment 4 suggested that many participants could have benefited from assistance on hearing aid use and care. Again, if questions were asked, it was recommended that participants review the hearing aid User Guide from the manufacturer. Fewer participants attended this appointment compared to the number of participants who attended Appointment 3. Some continued to report issues with hearing aid sound quality, inserting the hearing aids, and manipulating the hearing aid buttons. Again, several participants across groups had different hearing aid preset settings compared to the presets used in Appointment 2. Some participants, however, reported that they were satisfied or very satisfied with their hearing aids, especially those who were placed in the ABP+AR group (i.e., five participants). 

## 4. Discussion

This study has provided preliminary anecdotal comments and initial observations of hearing aid use and care from a group of 51 participants who completed four appointments associated with the OTC HA clinical trial taking place in five rural counties of West Central and South Alabama. The anecdotal comments and hearing aid settings observed during these appointments provided preliminary evidence that although some adults living in rural communities were capable of setting and maintaining their personal OTC HAs without assistance, others will most likely require support during initial hearing aid use periods. These finding have possible implications for how to support those with hearing loss in rural communities. 

The benefits received through OTC HA include a reduction in the negative consequences of hearing loss, including decreased social contact, loneliness, and declining cognitive skills [4,5,6,7]. Previous research has demonstrated that listening skills and perceived benefit from OTC HAs improve after receiving them. Specifically, outcomes from unaided to aided responses have demonstrated that hearing aids increase word understanding in noise after a short period of use [17,18,35]. Evidence also suggests that OTC HAs led to decreased negative social and emotional consequences of hearing loss [17,18]. However, it is important to note that adults in these studies had different demographic characteristics from those who participated in the current study. The group of adults in the current study were a heterogenous group living in rural communities, while those in mentioned previous studies were largely white, college educated, and lived in urban communities [17,18]. Different demographic characteristics might suggest the need for varying levels of support for adult populations in rural and urban communities. 

### 4.1. Addressing Individual Needs

Studies have demonstrated that, although individuals are capable of setting OTC HAs without professional support, satisfaction with the devices and performance outcomes were better when support was provided [17,18,36]. In these studies, most participants were able to set their devices independently, but those who were given support were more satisfied with them compared to those who were not given guidance. It was also noted that guidance at the beginning of hearing aid use and follow-up care could be critical to optimize success with OTC HAs [17,36]. In fact, a lack of counseling with hearing aid use has been shown to result in a lack of use [37]. 

Considering that there are few audiological resources in rural communities [9], new models for improving access to hearing healthcare are needed. Potentially, pharmacists or pharmacy technicians could be provided with basic training to help support and guide patients with purchasing OTC HAs in their rural community pharmacies. Future research will help to delineate the extent and type of training that would be needed to provide this basic care. 

### 4.2. Potential for Pharmacy and Audiology Collaboration

Evidence from our lab and from others has indicated that pharmacists and audiologists were excited by the potential for collaborating [11,13,14,16]. Specifically, Brothers and colleagues found that of the forty statements provided in focus group discussions and interviews with 15 pharmacists, only six statements expressed concern for the collaboration, which included issues associated with limited time and staff availability and prohibitive costs of the OTC HAs [14]. Midey and colleagues revealed that 94% of 85 pharmacists who responded to an online survey expressed interest in increasing their knowledge of OTC HAs, 72% expressed interest in selling OTC HAs in their pharmacies, and 73% expressed interest in assisting patients with the selection of OTC HAs [12]. Evidence also has suggested that pharmacists were capable of successfully providing OTC HAs to their patients [15]. In this study, a pharmacist, working in collaboration with an audiologist, confirmed the degree of hearing loss, ensured that contraindications for HA use were not present, assessed the patient’s perceived difficulty with their hearing loss, and helped with the setting of the initial HAs as well as a second set of aids after a malfunction of the first set. This case study suggested that pharmacists will need to have some understanding of hearing healthcare and work collaboratively with audiologists to provide appropriate care for their patients. Collectively, these studies provide the foundation for a meaningful collaboration between pharmacists and audiologists that has the potential to significantly improve access to hearing healthcare for those in underserved populations. 

## 5. Conclusions

Future outcomes from the listening tests and surveys that have been outlined in this OTC HA clinical trial, where participants were randomly placed in one of three support groups, will provide the foundation for understanding the extent of support needed for their successful use. Preliminary qualitative comments and observations indicate that although some adults were able to successfully set, use, and care for their devices, some required varying levels of assistance. These results could imply that adults in rural communities will require some guidance setting OTC HAs. Previous findings have indicated that improving access to hearing healthcare and OTC HAs in rural communities could occur through establishing collaborative initiatives between audiology and pharmacy providers.

## Figures and Tables

**Table 1:** Clinical Trial Schedule.

Week/s	Group Activities		
1 (Appt 1)	Hearing Evaluations and Questionnaires (Audiologists A1 and A2)		
	ABP+AR	ABP	OTC-only
2 (Appt 2)	OTC HAs provided by audiologist (A1) OTC HA Orientation	OTC HA provided by audiologist (A1) OTC HA Orientation	OTC HAs set independently No OTC HA Orientation
3 (Appt 3)	Listening Tests (A2)		
4–7	4 weeks AR Program	4 weeks General Health Sessions	4 weeks General Health Sessions
8 (Appt 4)	Follow-up Listening Testing and Questionnaires (A2)		
9 (Appt 5)	Optional Hearing Aid Check (A1 and A2)		
10–13	4 weeks General Health Sessions	4 weeks AR Program	4 weeks AR Program
14 (Appt 6)	Follow-up Listening Testing and Questionnaires (A3)		

Note: ABP = Audiological Best Practices: AR = Aural Rehabilitation (Hearing Coaching); OTC HA = Over-the-Counter Hearing Aids.

**Table 2:** Participant information.

Demographic	Total Count	Cumulative Total	ABP+AR Group	ABP Group	OTC-Only Group
Gender	*N* = 51	Percent	*N* = 17	*N* = 19	*N* = 15
Female	32	63%	10	12	10
Male	19	37%	7	7	5
Ethnicity					
Not Hispanic	51	100%	17	19	15
Hispanic	0	0%	0	0	0
Race					
Black/African American	27	53%	9	10	8
White	24	47%	8	9	7
Other	1	2%	0	1	0
Perceived Income Inadequacy					
Not Difficult at All	16	31%	4	6	6
Not Very Difficult	13	26%	4	6	3
Somewhat Difficult	17	33%	6	7	4
Very Difficult	5	10%	3	0	2
Education					
<High School	11	21%	4	2	5
High School or GED	14	27%	5	8	1
Some College	7	14%	3	2	2
2 year College Degree	5	10%	2	1	2
4 year College Degree	6	12%	1	1	4
Master’s Degree	7	14%	1	5	1
Doctoral Degree	1	2%	1	0	0

Note: One participant selected White and Other for race, which accounted for a Cumulative Total of more than 100%. Perceived Income Inadequacy was determined using the question, “How difficult is it for you to pay for the very basics, like food, housing, medical care, and heating?”. No participants reported being American Indian or Alaskan Native, Asian or Native Hawaiian, or Other Pacific Islander. ABP is Auditory Best Practices, AR is Aural Rehabilitation, and OTC is Over-the-Counter Hearing Aids.

**Table 3:** Anecdotal notes Appointment 2,setting OTC HAs.

Degree of Guidance	Notes
Independent *N* = 5	All used the small print version of the manual. No guidance was given to these participants.
Minimal Guidance *N* = 4	Three used the small print version of the instruction manual. One used the large print version of the instruction manual. The daughter of one participant read the instruction manual to their parent. Two needed guidance related to the selection of the dome size. Three needed guidance related to the preset instructions in the manual. One needed a demonstration of the power feature. One needed guidance related to the environmental listening mode.
Moderate Guidance *N* = 2	Two used the large print version of the manual. One needed guidance for the preset instructions. One needed to be shown the instructions in the manual for charging the hearing aids. For one, all the instructions in the manual, except the charging instructions, needed to be shown to them. One participant appeared to be frustrated and informed the audiologist she would read the manual on her own at home.
Full Guidance *N* = 4	Two used the large print version of the instruction manual. The two daughters of one participant read the manual to their parent. The daughter of one participant helped them set the device. The daughter of another participant was not able to assist their parent. All instructions for each hearing aid feature needed to be highlighted for all participants (i.e., dome selection, power, volume, preset, listening mode, and charging).

**Table 4:** Anecdotal notes from Appointment 3.

Group	Anecdotal Notes	Preset Change from Appt 2	HA Charged Status
ABP+AR *N* = 16	Powering on and off question, *N* = 1 Hearing aids set at different listening modes, *N* = 1 Cerumen buildup, *N* = 1 Background noise issues, *N* = 1 Static issues, *N* = 1 Asked about how long to use aids each day, *N* = 2 Not using aids, *N* = 1 OTC HA malfunction, *N* = 2 Needed indicator for the right and left ear, *N* = 2 No issues reported, *N* = 3	*N* = 4	Charged, *N* = 11 Not charged, *N* = 5
ABP *N* = 16	Not wearing them at appointment, *N* = 2 Unsure how to use buttons, *N* = 4 Aids reported to be too loud, *N* = 2 Dome help, *N* = 1 Using one aid, *N* = 1 OTA HA malfunction, *N* = 1 Placement issues, *N* = 3 No issues reported, *N* = 4	*N* = 4	Charged, *N* = 12 Not charged, *N* = 2
OTC-Only *N* = 13	Hearing aids on different listening modes, *N* = 1 Not using them regularly, *N* = 3 Using phone app, *N* = 1 Ear canal discomfort, *N* = 2 Placement issues, *N* = 3 Question about feedback, *N* = 1 Needed indicator for the right and left ear, *N* = 2 No issues reported, *N* = 4	*N* = 5	Charged, *N* = 11 Not charged, *N* = 2

**Table 5:** Anecdotal notes from Appointment 4.

Group	Anecdotal Notes	Preset Change from Appt 2	Charged Status
ABP+AR *N =* 14	Satisfied with hearing aids, *N =* 5 Not satisfied with hearing aids, *N =* 1 Not worn or not wearing consistently, *N =* 2 Hearing aid malfunction, *N =* 1 Issues with sound quality, *N =* 2 Issues inserting aids, *N =* 2 Using phone app, *N =* 3	*N =* 6	Charged, *N =* 13 Not charged, *N =* 1
ABP *N =* 15	Reported difficulty understanding others, *N =* 1 Reported significant benefit, *N =* 1 Reported difficulty controlling buttons, *N =* 2 Incorrect placement, *N =* 1	*N =* 5	Charged, *N =* 13 Not charged, *N =* 2
OTC-Only *N =* 12	Needed direction for charging aids, *N =* 1 Reported aids have not helped with tinnitus, *N =* 1 Reported aid sounded weak, *N =* 1 Using phone app, *N =* 2 Reported issues inserting aids, *N =* 1 Reported sound quality issues, *N =* 3 Hearing aid malfunction, *N =* 1 Not wearing consistently, *N =* 1	*N =* 4	Charged, *N =* 11 Not charged, *N =* 1

## Data Availability

The original contributions presented in the study are included in the article, and further inquiries can be directed to the corresponding author.
